# Vitamin K and Kidney Transplantation

**DOI:** 10.3390/nu12092717

**Published:** 2020-09-05

**Authors:** Maria Fusaro, Laura Cosmai, Pieter Evenepoel, Thomas L. Nickolas, Angela M. Cheung, Andrea Aghi, Giovanni Tripepi, Mario Plebani, Giorgio Iervasi, Roberto Vettor, Martina Zaninotto, Maura Ravera, Marina Foramitti, Sandro Giannini, Stefania Sella, Maurizio Gallieni

**Affiliations:** 1 National Research Council (CNR), Institute of Clinical Physiology (IFC), 56124 Pisa, Italy; iervasi@ifc.cnr.it; 2 Department of Medicine, University of Padova, 35128 Padova, Italy; roberto.vettor@unipd.it; 3 Nephrology Unit, ASST Fatebenefratelli Sacco, 20157 Milano, Italy; lacos@iol.it (L.C.); maurizio.gallieni@unimi.it (M.G.); 4 Laboratory of Nephrology, Department of Immunology and Microbiology, B-3000 Leuven, Belgium; pieter.evenepoel@uzleuven.be; 5 Division of Nephrology, Department of Medicine, Columbia University, New York City, NY 10032, USA; tln2001@cumc.columbia.edu; 6 Department of Medicine, University of Toronto, Toronto, ON M5S, Canada; angela.cheung@uhn.ca; 7 Department of Medicine, Clinica Medica 1, University of Padua, 35128 Padova, PD, Italy; andrea.aghi@gmail.com (A.A.); sandro.giannini@unipd.it (S.G.); stefania.sella@unipd.it (S.S.); 8 CNR-IFC, Clinical Epidemiology of Renal Diseases and Hypertension, Ospedali Riuniti, 89124 Reggio Calabria, Italy; gtripepi@ifc.cnr.it; 9 Laboratory Medicine Unit, Department of Medicine, University of Padua, 35128 Padova, Italy; mario.plebani@unipd.it (M.P.); marti.zaninotto@libero.it (M.Z.); 10 Policlinico San Martino, 16132 Genova, Italy; maura.ravera@hsanmartino.it; 11 Divisione di Nefrologia e Dialisi, Renal Department, ASST-Cremona, Largo Priori 1, 26100 Cremona, Italy; m.foramitti@asst-cremona.it; 12 Department of Biomedical and Clinical Sciences ‘Luigi Sacco’, Università di Milano, 20157 Milano, Italy

**Keywords:** kidney, transplant, phylloquinone, menaquinone, vitamin K, osteoporosis, bone, fracture, cardiovascular disease, calcification, cancer

## Abstract

The assessment of the vitamin K status and its effects on clinical outcomes in kidney transplantation (KT) patients has sparked interest, but it is still largely unfulfilled. In part, this is due to difficulties in laboratory measurements of vitamin K, especially K2 vitamers. Vitamin K status is currently best assessed by measuring undercarboxylated vitamin-K-dependent proteins. The relative contribution of vitamin K1 and K2 to the health status of the general population and CKD (chronic kidney disease) patients, including KT patients, is also poorly studied. Through a complete and first review of the existing literature, we summarize the current knowledge of vitamin K pathophysiology and its potential role in preventing KT complications and improving organ survival. A specific focus is placed on cardiovascular complications, bone fractures, and the relationship between vitamin K and cancer. Vitamin K deficiency could determine adverse outcomes, and KT patients should be better studied for vitamin K assessment and modalities of effective therapeutic approaches.

## 1. Introduction

Kidney transplantation (KT) has several significant benefits compared to dialysis treatment, such as a better quality of life, prolonged survival, and lower costs [1]. However, relevant clinical complications affect KT patients, including progressive decline in renal function [2,3], vascular calcifications (VCs) [4,5], and bone fractures (BFs) [6,7], which associate with a decreased survival of the graft and an increased mortality [5,7]. Unfortunately, the request for KT is higher than organ donations; therefore, it is essential to preserve graft survival as much as possible. A new therapeutic approach could be explored, Vitamin K. The aim of this review was to summarize the current vitamin K knowledge for its potential role in preventing KT complications and improving organ survival.

## 2. Vitamin K, a Family of Vitamers: Types, Status, and Vitamin-K-Dependent Proteins (VKDPs)

The 2-methyl-1,4-naphthoquinone group is common in K vitamers, including three main forms—phylloquinone (PK or vitamin K1), which can be found mostly in green vegetables and fruits; menaquinones (MKs or vitamin K2), classified conforming to the length of their unsaturated side chains (MK-4 to MK-15) [4]; and menadione (vitamin K3), a synthetic form. In humans, the most common K2 vitamer is MK-4, with the shortest chain, which is the only one produced by a systemic conversion from phylloquinone to menaquinones [8,9]. Intestinal bacteria synthesize vitamers MK-7 through MK-10. Additionally, they are also contained in meat, cheese, and fermented soy derivatives that use Bacillus Subtilis Natto, a traditional Japanese food (natto mainly contains MK7, and in smaller quantities, it contains MK8 and PK) [8,10]. Vitamin K is recycled continuously because there are very few vitamin K reserves [8]. 

Recommendations for daily intake of vitamin K are different in various countries because the data in the literature are scarce. In particular, in the United States, the Institute of Medicine has proposed an adequate intake (AI) for men and women of 120 and 90 μg/day, respectively. The Italian LARN (Reference Levels of Assumption of nutrients and energy) [11], has proposed a higher intake, stratified by age—140 or 170 μg/day for 18–59 and >60 years old, respectively. Furthermore, in 2016, the Belgian health authority (Conseil Supérieur de la Santé) issued a statement indicating a recommended daily intake of 50–70 μg/day of vitamin K1 for the adult population and a new increased maximal tolerable intake of vitamin K of 1 mg/day (0.017 μg/kg/day) [12]. Thus, a consensus on the definition of adequate intake for vitamin K does not exist, also because the contribution of the amount produced by intestinal bacteria remains uncertain. The absence of abnormal bleeding (an indication of physiological functioning of carboxylated coagulation factors) cannot be considered an estimate of adequate vitamin K levels, since it does not warrant vascular and bone health. 

More than 15 types of vitamin-K-Dependent Proteins (VKDPs) were identified until now. The most studied VKDPs are the following—several proteins affecting coagulation (protein C, S, M, Z, factors VII, IX, X, and prothrombin), bone Gla protein (BGP, or osteocalcin), matrix Gla protein (MGP), Gas6 (Growth Arrest-Specific 6 Protein), GRP (Gla Rich Protein), and periostin [13]. Vitamin K acts at least in two ways. First, as the coenzyme of a carboxylase, determining the carboxylation of glutamic acid residues with a resulting formation of the amino acid γ-carboxy-glutamic acid (Gla: active form) [13,14]. Second, as a ligand of the steroid and xenobiotic receptor (SXR) and pregnane X receptor (PXR, murine ortholog) [15]. SXR is a nuclear receptor involved in the transcriptional regulation of enzymes, such as cytochrome P450. Beyond being an inducer of detoxification and drug excretion genes, this receptor is expressed in osteoblasts and involved in bone metabolism [9,16]. Accordingly, PXR knockout mice develop an osteopenic phenotype [16]. 

Direct measurement of vitamers should be the ideal way to assess vitamin K status. However, to date, the standardization of the method is a great challenge. Obstacles are represented by lower physiological levels among fat-soluble vitamins and triglyceride interference, especially in chronic kidney disease (CKD) patients. Additionally, most studies only evaluated the PK levels (Table 1) [17,18]. On the other hand, the vitamin K status could be evaluated indirectly by the amount of undercarboxylated VKDPs (Table 1) [19,20,21].

Several factors can influence vitamin K status in CKD patients, either reducing or enhancing its activity (Table 2). Among the causes of vitamin K deficiency in CKD patients, the following are more common—dietary restrictions determining inadequate intake; dysbiosis due to the uremic condition that leads to a decreased vitamin K (especially long-chain MKs); production by microbiota; and hemodialysis associated deficits [8,22,23]. Treatment of mineral bone disease (MBD) in CKD can also reduce vitamin K and VKDPs’ actions. Some phosphate binders, such as sevelamer, bind fat-soluble vitamins, including vitamin K. Its use is associated with higher dp-ucMGP levels in KT patients [24]. In the VItamin K Italian (VIKI) study, a cross-sectional study of 387 hemodialysis patients, we established the prevalence of vitamin K deficiency. Additionally, we assessed the relationship between vitamin K status, vertebral fractures (VFs), and vascular calcifications (VCs). MK4 deficiency was the strongest predictor of aortic calcification (OR, 2.82; 95% CI, 1.14–7.01) [25]. In multivariable logistic regression, the odds ratio of MK4 deficiency in patients treated with sevelamer was 2.64, (95% CI: 1.25–5.58, *p* = 0.01) [26]. Furthermore, warfarin use interferes with vitamin K recycling, reducing γ-carboxylation, and consequently, the activity of VKDPs. Thus, warfarin-treated patients develop vitamin K deficiency, associated with a high prevalence of VCs, VFs (in males), and mortality, both in the general population [27,28] and in CKD patients [29,30].

On the other hand, in a secondary analysis of the VIKI study, we found that calcimimetics and vitamin D analogs might play a role in preserving VKDPs activity. We found an increase in total BGP levels and, only in patients treated with calcimimetics, the total MGP levels increased [31]. Furthermore, Keyzer et al. showed in 518 KT recipients with 6-year follow-up of an inverse association between dp-ucMGP levels and mycophenolate mofetil (MMF) use [5], confirming a previous finding of an association between thoracic aorta calcification and shorter time on MMF treatment [32]. A recent prospective study of 34 patients assessed changes in VKDPs during the 1st year of KT, showing a decrease in the undercarboxylated (inactive form) amount [33].

### 2.1. Vitamin K and Cardiovascular Disease in Kidney Transplantation

**Figure 1:**
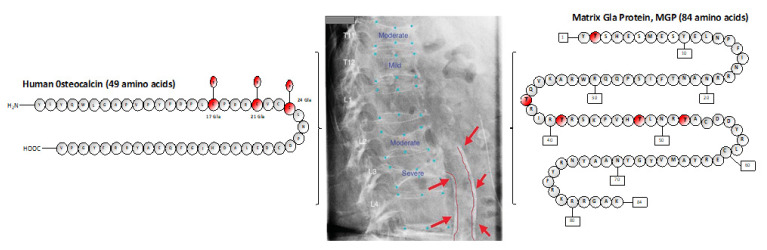
The two better-studied vitamin-K-dependent proteins, bone Gla protein (BGP, osteocalcin) and matrix Gla protein (MGP), contribute to cardiovascular and bone health in the general population and kidney transplant patients, who have an increased risk of cardiovascular events and bone fractures. Aortic calcifications and vertebral fractures are typical complications seen in CKD patients.

Cardiovascular disease, strongly associated with vascular calcification, is the leading cause of death in end-stage kidney disease. Vascular calcification is inhibited by the vitamin-K-dependent protein MGP (matrix γ-carboxy-glutamic acid protein), which requires serine phosphorylation for its activation, beyond the gamma-carboxylation [13] (Figure 1). Thus, assessing the role of vitamin K in the complex setting of KT is of interest. Vascular calcifications, which can be linked to abnormalities in vitamin K status, are potential factors affecting cardiovascular health in transplanted patients.

Jansz et al. [24] measured plasma dp-ucMGP, a marker reflecting low vitamin K status, in a cross-sectional study of 113 dialysis and 36 KT patients. Dp-ucMGP levels were significantly lower in KT recipients (median 689 pmol/L), compared to patients on dialysis (median 1537 pmol/L, *p* < 0.001) [24]. These results suggest an improved vitamin K status after KT, which might lower the risk of developing vascular calcification. Preliminary data from Fusaro et al. [33] confirmed that serum concentrations of inactive vitamin-K-dependent proteins decrease 1-year post kidney transplantation, including dp-ucMGP (median pre-KT 910 pmol/L, post-KT 637 pmol/L, *p* < 0.001). The prevalence of vitamin K deficiency, defined by the cut-off level of uc-BGP >4.5 ng/mL, decreased from 76.5% to 32.4% after KT. 

Lees et al. [34] performed a meta-analysis of studies investigating vitamin K status, supplementation, and vascular disease. Specifically, they studied the effect of vitamin K supplementation on vascular stiffness and vascular calcification, as well as the association of inactive VKDP levels with incident cardiovascular disease and mortality. Combining data in different patient populations from 13 controlled clinical trials and 14 longitudinal studies (with substantial heterogeneity), supplementation with vitamin K significantly reduced vascular calcification, but not stiffness. Additionally, inactive VKDP levels (dp-ucMGP and ucBGP, indicating a vitamin K deficiency status) were associated with the combined endpoint of cardiovascular diseases or mortality. 

Another meta-analysis, including 21 studies on different population cohorts totaling over 200,000 patients [35], pointed out an association of vitamin K with cardiovascular events and all-cause mortality. Higher dietary vitamin K consumption was associated with a significantly lower risk of coronary artery disease. For vitamin K1, the risk reduction was 8% (HR 0.92, 95% CI 0.84–0.99), and for vitamin K2, risk reduction was 30% (HR 0.70, 95% CI 0.53–0.93). Higher levels of dp-ucMGP were associated with increased risks (pooled HR 1.84) of all-cause and cardiovascular mortality, although the analysis could not establish causal relations. 

Focusing on the KT population, Mansour et al. [36] investigated the association between the change in vitamin K status and indices of arterial stiffness in 60 patients, following eight weeks of menaquinone-7 (vitamin K2) supplementation (360 μg once daily). They found that 53% of patients had a subclinical vitamin K deficiency. Menaquinone-7 supplementation reduced the mean dp-ucMGP concentrations by 55% and the prevalence of subclinical vitamin K deficiency by 40%. In addition, an improvement in arterial stiffness was independently associated with the reduction in dp-ucMGP concentration.

Data on the association between vitamin K status and mortality after KT were also available from a single-center observational study in 518 patients [5]. Most patients (91%) had vitamin K insufficiency, defined by serum dp-ucMGP levels >500 pmol/L. Patients in the highest quartile of dp-ucMGP had a higher mortality risk than patients in the lowest quartile (HR, 3.10; 95% CI, 1.87–5.12). This observational study indicated the opportunity of a randomized trial investigating whether vitamin K supplementation might lead to improved outcomes after KT [5]. Accordingly, the same group is now conducting a study [37] in KT recipients with vitamin K deficiency, whose main objective is to investigate the effect of vitamin K2 supplementation on serum calcification propensity. Calcification propensity is a surrogate marker, assessing the tendency to develop future vascular calcifications. The patient’s serum is challenged with supersaturated calcium and phosphate solutions, which leads to the formation of primary calciprotein particles (CPPs). Such particles are then transformed into secondary CPPs with different timing, depending on the balance between calcification promoters and inhibitors. Secondary endpoints of the study include changes from the baseline in dp-ucMGP levels and vascular stiffness. 

In a study aimed at assessing the prevalence and determinants of vascular calcifications in 281 renal transplant recipients, Nguyen et al. [32] found, among the determinants of aortic calcification, a role for a shorter exposure to mycophenolate mofetil and the current use of warfarin. Interestingly, Boxma et al. [38] found that dp-ucMGP levels were higher in patients on calcineurin inhibitors (855 (590–1350) pmol/L), indicating vitamin K deficiency, and were lower in patients on mycophenolate mofetil (591 (479–897) pmol/L). Thus, the protective effects of mycophenolate mofetil against vascular calcification [32] might be at least in part mediated by a favorable effect on vitamin K metabolism. 

Preliminary data of a randomized, double-blind, placebo-controlled trial of vitamin K supplementation to improve vascular health in a small and heterogeneous cohort of 90 prevalent kidney transplant recipients, were recently presented and published in abstract form [39]. Patients were treated with vitamin K (menadiol diphosphate 5 mg) or placebo, thrice weekly for one year. The primary outcome was between-group differences in vascular stiffness at one year. Coronary calcifications were among the secondary outcomes. Vitamin K supplementation did not reduce vascular stiffness or calcification over one year. Future studies should consider larger sample sizes, higher doses, and a different combination of vitamin K1 and K2, considering that menadiol diphosphate, used in this study, is a synthetic form of vitamin K, which might act differently from vitamin K1 and K2 on vascular health.

Table 3 summarizes the main studies [4,5,7,24,36,38] that evaluated the vitamin K status in organ transplantation. Another study in lung and heart transplantation demonstrated that bone status improved in patients with low vitamin K2 levels, after dietary supplementation [40]. 

### 2.2. Vitamin K and Bone Fractures in Kidney Transplantation

Bone fractures (BFs) are relevant MBD complications among CKD patients. In particular, hip fractures are four times more common than the general population [41,42]. In KT patients, there is a 30% increase of BFs in the first three years after transplantation [7]. 

Vitamin K can influence bone health in different ways. First, carboxylation of glutamine residues at 17, 21, and 24 of bone Gla protein (carboxylated BGP:cBGP, active form) allows its binding to bone hydroxyapatite, so that cBGP can exercise both an inhibition of bone mineralization and the regulation of the rate of mineral maturation [43]. Accordingly, BGP knockout mice develop hyperostosis [44]. Second, vitamin K2 (vitamin K1 is not capable of activating SXR until after its conversion into MK-4) acting as a ligand of SXR/PXR induce the transcription of several genes relevant to mineral metabolism—genes encoding bone collagen proteins (Tsukushi, Matrilin-2, primary SXR target genes) affecting bone quality, and genes regulating osteoblastogenesis, osteoclastogenesis (CD14), and osteoblast differentiation (Msx2) [16,45] (Figure 1).

Furthermore, beyond the well-known protective effects of MGP in vascular calcification, a recent study [46] highlighted its role in skeletal health, indicating that MGP overexpression suppresses osteoclast differentiation and bone resorption. In fact, the expression of the Nuclear Factor of Activated T cells, cytoplasmic 1 (NFATc1), which is the leading player in osteoclastogenesis, is controlled by MGP [46,47,48]. These experimental data were supported by the clinical results obtained in 468 de novo renal transplant recipients [7]. In this cohort, Cox proportional hazards analysis found that a dp-ucMGP above the median (1150 nmol/L, and notably 421 patients had levels >500 nmol/L, indicating a condition of vitamin K deficiency) was associated with incident fractures (HR 2.21, *p* < 0.05). Moreover, high dp-ucMGP levels were independently associated with elevated inflammatory markers and low BMD [7]. 

Several studies showed a relationship between low vitamin K intake or low vitamin K levels, and fracture risk in the general population. In the VIKI study [25], we highlighted that Vitamin K1 deficiency was the strongest predictor of vertebral fractures (VFs) (OR: 2.94, *p* = 0.0053) in 387 HD patients. Cheung et al. confirmed our findings in a study of 440 postmenopausal women. They gave 5 mg of vitamin K1 daily for two years, showing a reduction of the risk of VFs (HR 0.45, *p* = 0.04), despite a lack of effects on BMD and bone resorption, sustaining the hypothesis of a potential role of vitamin K in preserving bone quality [49]. Furthermore, Menatetrenone or MK-4 was approved as a drug for the treatment of osteoporosis in Japan in 1960. Indeed interventional studies with MK-4 (45 mg/day orally) demonstrated both a reduction of the incidence of bone fractures and improvements of BMD [50]. 

### 2.3. Vitamin K and Cancer in Kidney Transplantation 

After cardiovascular diseases, cancer is the second most common cause of mortality and morbidity in KT recipients, showing at least a twofold higher risk of developing cancer or dying from cancer than the general population. The increased cancer risk in transplant recipients is multifactorial and is attributed to oncogenic viruses, immunosuppression, altered T cell immunity, or a combination of the above [51]. The standardized incidence ratios (SIRs) for infection-related malignancies—i.e., Epstein—Barr virus (EBV)-associated lymphoma, Kaposi sarcoma (KS), hepatocellular carcinoma, genital, and gastric cancers—are significantly elevated in kidney transplant recipients. However, a number of other cancer types unrelated to infection are also more common in the transplanted population, including squamous cell cancers of the skin and lip, renal cell carcinoma (RCC), cholangiocarcinoma, and salivary gland cancer. Moreover, the incidence of lung and colorectal cancer is even higher in the transplant population. In contrast, the SIR of prostate and breast cancer is not higher in transplant recipients compared to the general population [52]. 

There are no studies correlating the vitamin K nutritional status in KT patients with the incidence of post-transplant cancers. However, it is reasonable to think that the vitamin K status might also be involved in the development of cancer, as an additional risk factor, because abnormalities of several VKDPs are related to cancer.

### 2.4. Growth Arrest-Specific Protein 6 (Gas6)

Gas6 is a human gene encoding the Gas6 protein, present in several tissues (e.g., vascular endothelium, kidney, heart, and the bone marrow) and is upregulated in growth-arrested fibroblasts [13]. Gas6 shows the highest affinity for Axl, followed by Tyro3 and then for Mer (TAM) receptors, leading to the activation of downstream signaling, such as phosphatidylinositol 3-kinase (PI3K), extracellular signal-regulated kinase (ERK), and nuclear factor kappa-light-chain-enhancer of activated B cells (NF-kB) pathways. Thus, it is involved in the stimulation of cell proliferation, migration, differentiation, adhesion, and apoptosis [53]. 

Upregulation of Gas/TAM can promote the development of several cancers, and clinically the expression of Gas6 and TAM receptors predicts a poor prognosis. Specifically, Gas6 is overexpressed in melanoma, schwannoma, glioma, and pancreatic ductal adenocarcinoma cell lines, and it is upregulated in ovarian cancer and thyroid cancer specimens [54]. Furthermore, one study showed that Gas6 is amplified in breast cancer, and human prostate cancer cell lines were found to grow significantly better in vertebral bodies transplanted from Gas6-/- animals than in those derived from Gas6 +/+ animals [55,56]. 

Gas6 is also highly expressed in bladder cancer. It is significantly associated with tumor grade, T stage, and worse prognosis. GAS6 might play a pivotal role in the development of bladder cancer, being a potential target for its treatment [57].

### 2.5. Periostin

Similar to other VKDPs, the carboxylation of periostin is dependent on vitamin K. Periostin is an extracellular matrix protein that binds integrins, leading to stimulation of cellular adhesion and migration [58].

The tumor microenvironment is highly complex, and consists of non-tumor cells, extracellular matrix proteins (matricellular proteins), and soluble factors, modulating the immune response against tumor cells [59].

Periostin is a matricellular protein, and excessive periostin deposition plays a pivotal role in cancer cell proliferation, invasion, and dissemination [60]. In breast cancer, periostin is expressed in invasive ductal carcinoma cells [61], and it might play a role in cancer progression [62]. Periostin could be a marker of breast cancer metastasis [63]. Finally, periostin could predict prognosis in patients with breast cancer. In breast cancer patients who underwent surgery and radiation therapy, local recurrence-free survival, distant metastasis-free survival, and overall survival were significantly lower in the patients whose tumors expressed periostin [64].

Serum periostin levels were significantly higher in patients with colorectal cancer (CRC) than in healthy controls and were associated with clinical stages. Patients with lower serum periostin had better survival than those with high serum periostin [65]. A study of resected hepatocellular carcinoma (HCC) specimens showed that high periostin levels were more frequent in cases of multiple tumors, presence of microvascular invasion, and advanced-stage disease. Furthermore, patients with high periostin expression had significantly lower overall survival rates than those with low periostin expression [66]. As a whole, periostin is highly expressed in a large number of cancers, and this association confers a worse prognosis to patients, as shown in Table 4.

### 2.6. Vitamin K Administration in Cancer Prevention and Treatment

Several VKDPs are involved in cancer development. Vitamin K2 administration in vivo inhibits the cellular proliferation of several types of cancers [72,73]. Many studies investigated the role of vitamin K intake and supplementation in the prevention of cancer development, progression, and recurrence [74]. 

The European Prospective Investigation into Cancer and Nutrition Heidelberg cohort study, which included 24,340 cancer-free participants followed up for ten years, found a significant inverse association between vitamin K2 intake and cancer mortality, but not cancer incidence [75]. However, a significant inverse relationship between cancer incidence and Vitamin K2 intake was demonstrated in men [75]. In the Prevención con Dieta Mediterránea study (PREDIMED), with a median follow-up of 4.8 years, the dietary intake of both vitamin K1 and K2 was associated with a decreased cancer incidence [76]. Notably, vitamin K intake was substantially higher than that in the Heidelberg cohort [76].

Vitamin K2 supplementation in patients who underwent curative hepatectomy or radiofrequency ablation for HCC, reduced HCC recurrence, although not significantly [77,78]. Additionally, 45 mg per day of vitamin K2 supplementation resulted in a significantly lower risk of HCC development in women with viral cirrhosis [79], suggesting that vitamin K2 might play a role in preventing the development of HCC in high-risk patients [79]. 

As a whole, the association of Vitamin K with cancer is still under investigation, the research suggests that vitamin K is involved in anti-tumor responses through several mechanisms. Whether a suboptimal vitamin K status contributes to cancer development is not yet established; further studies will hopefully define the role of vitamin K, especially in transplanted patients.

## 3. Conclusions

After kidney transplantation, vitamin K status improves compared to patients remaining in dialysis care, and the progression of calcification slows down. However, because bone fractures and vascular calcifications remain a significant cause of morbidity and mortality in kidney transplant recipients, more evidence is undoubtedly needed on the effects of vitamin K supplementation at physiologic and pharmacologic doses of different vitamers of the vitamin K family.

## Figures and Tables

**Table 1:** Levels of molecules defining vitamin K deficiency.

Molecule	Direct Measurement	Indirect Measurement
PK	General population: <0.3 nmol/L [17] CKD patients <0.4 nmol/L [18]	/
MKs	Uncertain	/
PIVKA	/	>2 nmol/L [19]
ucBGP	/	>20% or ≥4.5 ng/mL [19,20]
dp-ucMGP	/	>500 pmol/L [21]

Abbreviations: PK—phylloquinone; MKs—menaquinones; PIVKA—protein induced by vitamin K absence; ucBGP—undercarboxylated BGP; and dp-ucMGP—dephosphorylated-undercarboxylated MGP. CKD: chronic kidney disease.

**Table 2:** Factors affecting vitamin K status in chronic kidney disease (CKD) patients.

Increase in VKDPs Activity	Decrease in VKDPs Activity
Calcimimetics use [31]	Poor vitamin K intake [22]
Vitamin D analogs use [31]	Dysbiosis due to the uremic condition [7]
MMF use [5,32]	Hemodialysis treatment [23]
Kidney transplantation [33]	Sevelamer use [24,26]
	Warfarin use [25]

VKDPs: Vitamin-K-Dependent Proteins, MMF: mycophenolate mofetil.

**Table 3:** Vitamin K status in kidney transplantation studies.

Author, Year	Patient Number	% of Patients with Vitamin K Deficiency	VKDP Measured
Boxma, 2012 [38]	60	80%	dp-ucMGP
Keyzer, 2015 [5]	518	91%	dp-ucMGP
Mansour, 2017 [36]	60	53.3%	dp-ucMGP
Jansz, 2018 [24]	32	62%.	dp-ucMGP
Evenepoel, 2019 [7]	468	90%	dp-ucMGP
van Ballegooijen, 2020 [4]	461	50%	dp-ucMGP

VKDP: Vitamin-K-Dependent Proteins, dp-ucMGP: dephosphorylated-undercarboxylated MGP.

**Table 4:** Periostin overexpression and cancer prognosis.

Site of Expression	Cancer	Cancer Outcome
Stroma	Prostate cancer [67] Lung cancer [68] Colorectal cancer [69] Breast cancer [62] Bladder cancer [70] Hepatocellular cancer [71] Pancreatic cancer [59] Ovarian cancer [59]	Poor prognosis Reduced OS Reduced PFS Advanced stage and metastasis
Cancer epithelial cells	Colorectal cancer [69] Breast cancer [63] Hepatocellular cancer [71] Pancreatic cancer [59] Ovarian cancer [59]	Reduced OS Reduced PFS Tumor grade (poor prognosis) Increased microvascular invasion (poor prognosis) Advanced stages and cancer recurrence
Cancer-associated fibroblast	Breast cancer [62]	Reduced OS Reduced PFS
Extracellular vesicles	Bladder Cancer [70]	Tumor stage (poor prognosis)
Tumor	Osteosarcoma [59]	Reduced OS Reduced PFS

OS—Overall Survival; PFS—Progression Free Survival.
